# Heat Shock Response Regulator Is Pinned to the Membrane

**DOI:** 10.1371/journal.pbio.1001736

**Published:** 2013-12-17

**Authors:** Richard Robinson

**Affiliations:** Freelance Science Writer, Sherborn, Massachusetts, United States of America

Heat is the enemy of stability, and nowhere is this more true than in living cells, whose delicately folded proteins fall apart as temperatures rise. To combat this crisis, cells mount a “heat shock response,” a central feature of which is the coordinated production of chaperone proteins, to refold proteins that can be salvaged, and proteases, to degrade those that can't. In the *Escherichia coli* bacterium, the heat shock response is coordinated by sigma-32, a protein that binds to RNA polymerase and directs it to the promoters of chaperone and protease genes. Much is known about the regulation of sigma-32, including a variety of positive and negative feedback loops that govern its expression and activity (increasing free chaperones, for instance, reduces sigma-32 expression). But it has been clear from some disparities between prediction and reality that some critical element of sigma-32 activity has remained elusive. In a new study in *PLOS Biology*, Bentley Lim, Carol Gross, and colleagues pin down that missing element, showing that, rather than being dispersed in the cytoplasm as previous work had assumed, sigma-32 must be tethered to the bacterial membrane to be properly regulated.

**Figure:**
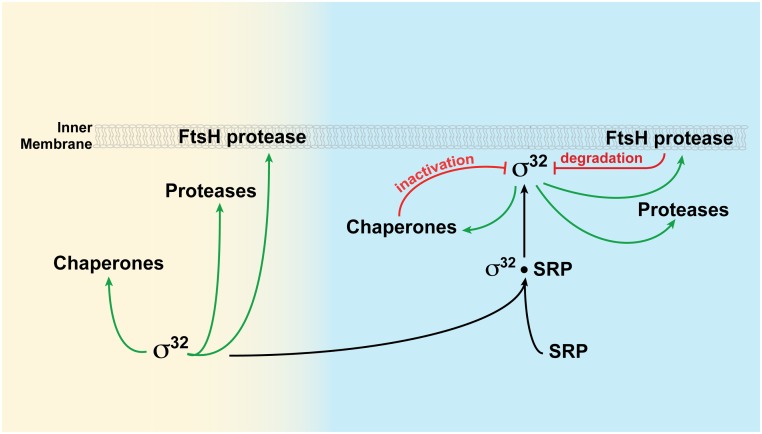
The σ^32^ protein can promote transcription of chaperones and proteases while in the cytosol or associated with the inner membrane, but is properly regulated only when membrane associated. Green arrows denote transcription; red arrows denote negative regulation.

The authors began by performing a genetic screen for new regulators of sigma-32. They found that reduction in expression of the signal recognition particle (SRP) receptor increased the activity of sigma-32, but also prevented its normal inhibition by chaperones. They used immunoprecipitation to show that sigma-32 co-precipitated with SRP, and gel filtration to show a direct interaction. These results immediately implied that sigma-32 localized to the inner bacterial membrane, since the function of SRP and its receptor is to target proteins there. The interaction of sigma-32 with SRP, and its localization to the membrane, could be reduced by mutation of sigma-32's so-called homeostatic control region, a small cluster of amino acids known to be critical for its regulation, further implicating membrane localization via SRP as a key to normal sigma-32 function. Finally, they artificially tethered a mutant in the homeostatic control region to the inner membrane using a viral coat protein. This single modification was able to confer full regulatory control of the sigma-32 heat shock response, including responsiveness to elevation of chaperones, even when SRP and the SRP receptor levels were reduced.

The placement of sigma-32 at the inner membrane may allow it to sense the state of protein folding both in the cytoplasm and in the membrane, the authors argue. The SRP system targets a wide variety of proteins to the membrane, and is thought to minimize protein misfolding by making translation coincident with membrane insertion. The SRP-dependent presence of sigma-32 at the membrane may further reduce or mitigate the potential for accumulation of misfolded proteins during translation through its control of chaperones and proteases.

**Lim B, Miyazaki R, Neher S, Siegele DA, Ito K, et al. (2013) Heat Shock Transcription Factor σ^32^ Co-opts the Signal Recognition Particle to Regulate Protein Homeostasis in** ***E. coli*** **.** doi:10.1371/journal.pbio.1001735

